# Scaffold-free self-assembled cell ring organoids enable efficient tumor modeling with high engraftment rates, superior tumor uniformity, and reduced animal consumption

**DOI:** 10.1016/j.mtbio.2026.103790

**Published:** 2026-11-02

**Authors:** Zhicheng Tong, Shuo Yang, Jiayi Chen, Haidong Guo, Piqian Zhao, Hongwei Wu

**Affiliations:** a Department of Orthopedics, Center for Regeneration and Aging Medicine, The Fourth Affiliated Hospital of School of Medicine, and International School of Medicine, International Institutes of Medicine, Zhejiang University, Yiwu, 322000, China; b Department of Orthopedics, Hunan Cancer Hospital and the Affiliated Cancer Hospital of Xiangya School of Medicine, Central South University, Changsha, 410013, China

**Keywords:** Cell ring organoids, Scaffold-free self-assembly, Patient-derived xenograft model, Tumor microenvironment, 3R principles

## Abstract

**Background:**

Conventional tumor cell suspension xenografts suffer from low engraftment efficiency, prolonged latency, and poor tumor uniformity, with high animal consumption conflicting with the 3R principles (Replacement, Reduction, Refinement). This study aimed to develop a scaffold-free self-assembled cell ring organoid platform to address these bottlenecks.

**Methods:**

Tumor cells and fibroblasts were mixed at optimized ratios and seeded into microwell molds, spontaneously self-assembling into ring-shaped organoids within 24–48 h without exogenous matrices or hydrogels. *In vitro* characterization included live/dead staining, histology, immunofluorescence, flow cytometry, Western blotting, and transcriptomic sequencing. *In vivo* performance was evaluated by orthotopic transplantation into the lateral femoral condyle of immunodeficient or immunocompetent mice, with longitudinal monitoring by bioluminescence imaging and micro-CT.

**Results:**

Cell rings maintained high viability during *in vitro* culture and were compatible with both cell lines and clinical primary sarcoma cells. Immunofluorescence and Western blotting revealed that co-cultured fibroblasts underwent cancer-associated fibroblast (CAF)-like phenotypic transformation (α-SMA upregulation) within the cell ring organoids. Transcriptomic analysis further demonstrated that the gene expression profiles of cell ring organoids closely resembled those of original tumors, whereas 2D-cultured cells showed the greatest number and magnitude of differentially expressed genes. *In vivo*, cell ring organoids achieved higher engraftment rates, better tumor uniformity, and stronger bone invasion capacity compared with cell suspensions.

**Conclusion:**

The scaffold-free self-assembled cell ring organoids platform provides an efficient, uniform, and 3R-compliant strategy for animal tumor modeling. Without requiring exogenous matrices, this technology achieves significantly higher engraftment rates and tumor uniformity compared with conventional methods, effectively reduces animal consumption, and may offer a potential platform for future preclinical drug screening and tumor microenvironment (TME) research.

## Graphical abstract

**Figure:**
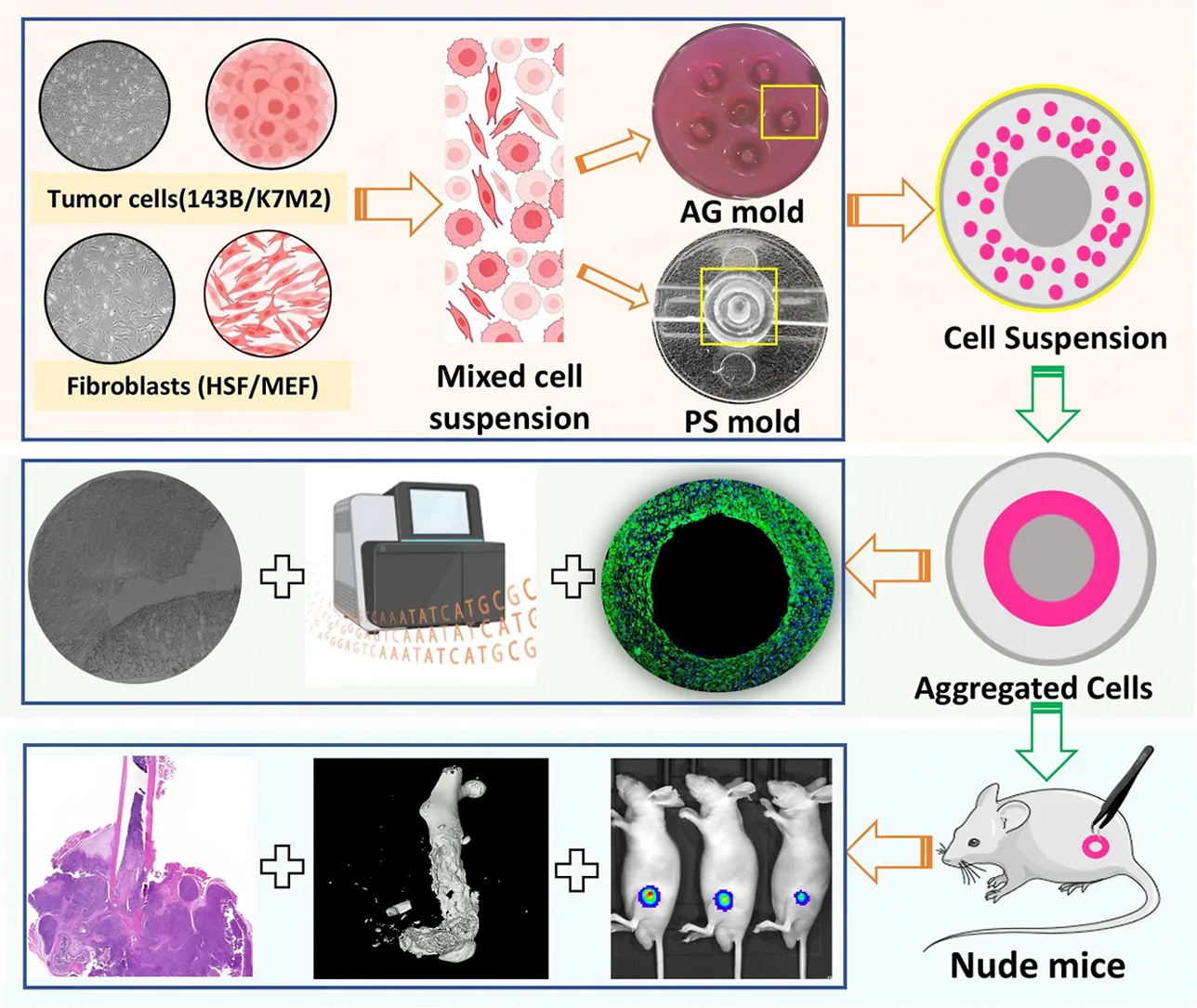

## 1 Introduction

Preclinical osteosarcoma research relies heavily on animal tumor models to evaluate the *in vivo* therapeutic efficacy and biological safety of anti-tumor agents, serving as the core bridge between fundamental tumor mechanism studies and clinical translation. To recapitulate the key disease features of osteosarcoma—including primary tumor growth, bone invasion, and pulmonary metastasis—researchers have developed various established mouse modeling approaches, encompassing three major categories: cell suspension injection models, genetically engineered mouse models (GEMMs), and patient-derived xenograft (PDX) models.

Each of these conventional osteosarcoma mouse models has unavoidable limitations (summarized in Table 1). Cell suspension injection represents the most widely used approach; however, orthotopic intratibial injection frequently causes tumor cell embolization [1], [2]. Perimandibular orthotopic models yield uniform tumor growth, but ethical regulations restrict the maximum allowable jaw tumor volume [3]. Tail vein injection of LM8 or SAOS-LM6 cells can model pulmonary metastasis, yet the procedure is complex and lung colonization efficiency is inconsistent [4], [5], [6], [7]. Intratibial injection of human tumor cells into SCID mice enables rapid tumor formation and early lung metastasis, but the immunodeficient host precludes investigation of the intact tumor immune microenvironment [8]. Subcutaneous transplantation of 3D GelMA microsphere-encapsulated K7M2 cells better preserves the original biological properties of tumor cells, though the preparation and transplantation procedures are cumbersome [9]. GEMMs faithfully recapitulate the core pathological features of human osteosarcoma; however, these models depend on customized transgenic mouse line construction, imposing a high experimental barrier [10], [11], [12]. The WWOX/p53 double-knockout GEMM exhibits enhanced tumor aggressiveness, yet the molecular mechanism by which WWOX loss facilitates genomic instability *in vivo* remains unverified [13]. GEMMs overexpressing the Notch1 intracellular domain develop multifocal osteosarcoma but also induce systemic osteosclerotic lesions, confounding independent tumor phenotype assessment [14]. PDX models preserve the histological characteristics of clinical osteosarcoma specimens, though their prolonged establishment cycle cannot meet the throughput demands of drug screening [15].

**Table 1:** Comparison of mouse models of osteosarcoma: Types, characteristics, advantages and limitations.

Model	Description	Mouse genotype or background	Tumor location	Advantages	Disadvantages	Reference
Cell suspension injection	K7M2 injected into the tibia of mice	BALB/c mice	Tibia	◆ A high likelihood of direct seeding of the pulmonary vascular system	◆ Embolization of large groups of tumor cells	[1], [2]
Cell suspension injection	0.50 × 10^6^ MOS-J cells injected to perimandibular sites	C57Bl/6	Jaw	◆ The tumor grows relatively uniformly	◆ Ethical constraints limit maximal jaw tumor size	[3]
Cell suspension injection	LM8 cells injected into the tail vein of the mice	CH3	Lung	◆ Higher incidence of pulmonary metastasis	◆ A rather complicated process	[4], [5]
Cell suspension injection	i.v. Injection of 10^6^ SAOS-LM6 cells	Athymic nude mice	Lung	◆ The superior ability of SAOS-LM6 cells to colonize the lung	◆ A rather complicated process	[6], [7]
Cell suspension injection	G-292 or HOS cells injected into the proximal tibial metaphysis	SCID mice	Proximal tibia	◆ The tumor grows rapidly and develops lung metastasis at an earlier stage.	◆ Unable to study the tumor immune microenvironment	[8]
Cell suspension injection	K7M2 cultured in 3D GelMA microspheres (1 × 10^6^ cells/mouse), which implanted subcutaneously into mice	BALB/c mice	Axilla	◆ Preferably maintain their original properties	◆ A rather complicated process	[9]
GEMM	Osteoblast-restricted deletion of p53 and pRb	Osx^−^Cre ^+^ p53^fl/fl^pRb^fl/fl^ (Osterix-Cre transgenic mouse)	Jaw and head	◆ Exhibite many of the features characteristic of human OS	◆ Requires transgenic mouse	[10], [11], [12]
GEMM	The Wwox and p53^Δosx1^ double knockout	Wwox/p53^ΔOsx1^ mice	Vertebrae, limbs, hip and jaw	◆ The tumors are more aggressive	◆ WWOX loss-mediated *in vivo* genomic instability unconfirmed	[13]
GEMM	Conditional expression of the intracellular domain of Notch1 (NICD)	NICD	Limb bones, rib, hip, spine and skull	◆ The tumors are more aggressive	◆ Exhibit generalized osteosclerotic disease	[14]
The patient-derived xenograft (PDX) model	The transplantation of freshly human osteosarcoma tissue into immunocompromised mice	BALB/c mice	Flank region	◆ The PDX model maintained the histology of the original human osteosarcoma tissues.	◆ The establishment cycle of the PDX model is long	[15]

None of the above mainstream models simultaneously achieves high engraftment rates, uniform tumor growth, and reduced animal consumption. Engraftment success rates are inconsistent, and intra-group tumor growth variability is substantial, such that effective experiments often require large numbers of mice. This conflicts with the “Replacement, Reduction, Refinement” (3R) ethical principles advocated in current animal research [16], [17], severely constraining standardized *in vivo* investigations of osteosarcoma invasion, metastasis, and targeted therapeutics. There is an urgent need for the development of novel standardized modeling approaches.

In recent years, 3D organoid culture technologies have advanced rapidly, offering new paradigms for tumor modeling and drug screening [18], [19]. Compared with traditional 2D monolayer culture, organoids more faithfully recapitulate the spatial architecture, gene expression profiles, and biological functions of native tumors [20], [21]. Current mainstream 3D tumor modeling approaches fall into two categories: organoid culture systems supported by exogenous matrices such as Matrigel or collagen, and xenograft protocols involving direct injection of dissociated single-cell suspensions. Both approaches have significant limitations. Matrix components are complex with poor batch-to-batch stability, and mechanical parameters are difficult to standardize, substantially compromising experimental reproducibility; moreover, residual matrix may trigger local inflammatory responses in animals, confounding tumor microenvironment (TME) evaluation [22], [23]. Single-cell suspension transplantation disrupts native cell–cell and cell–matrix interactions, rendering transplanted cells vulnerable to anoikis and fluid-mediated clearance, ultimately resulting in low engraftment rates, variable latency, and marked intra-group tumor heterogeneity [24], [25]. In summary, existing modeling approaches cannot simultaneously achieve 3D structural integrity, experimental standardization, and efficient *in vivo* transplantation. Developing a novel organoid transplantation platform that requires no exogenous matrix and can preserve intact cell interaction structures thus represents an urgent unmet need in the field of orthopedic oncology animal models.

To address these challenges, we developed a scaffold-free self-assembled cell ring organoid-based tumor modeling approach, with the complete technical workflow illustrated in Fig. 1. This technology uses microwell molds to guide tumor cells and fibroblasts to spontaneously self-assemble into ring-shaped organoids supported by endogenous extracellular matrix (ECM) within 24–48 h, entirely without exogenous matrices or hydrogels [26], [27]. These pre-formed toroidal constructs possess the following notable features: (1) they preserve the cell–cell and cell–matrix interactions characteristic of 3D tissues, obviating the need for post-transplantation re-aggregation and substantially reducing the risk of cell death; (2) the ring geometry facilitates inward nutrient diffusion, and the central lumen can rapidly interface with host vasculature after transplantation; (3) the co-cultured fibroblasts provide essential stromal support and paracrine signals to tumor cells, effectively reconstituting key components of the TME [28], [29].

**Fig. 1:**
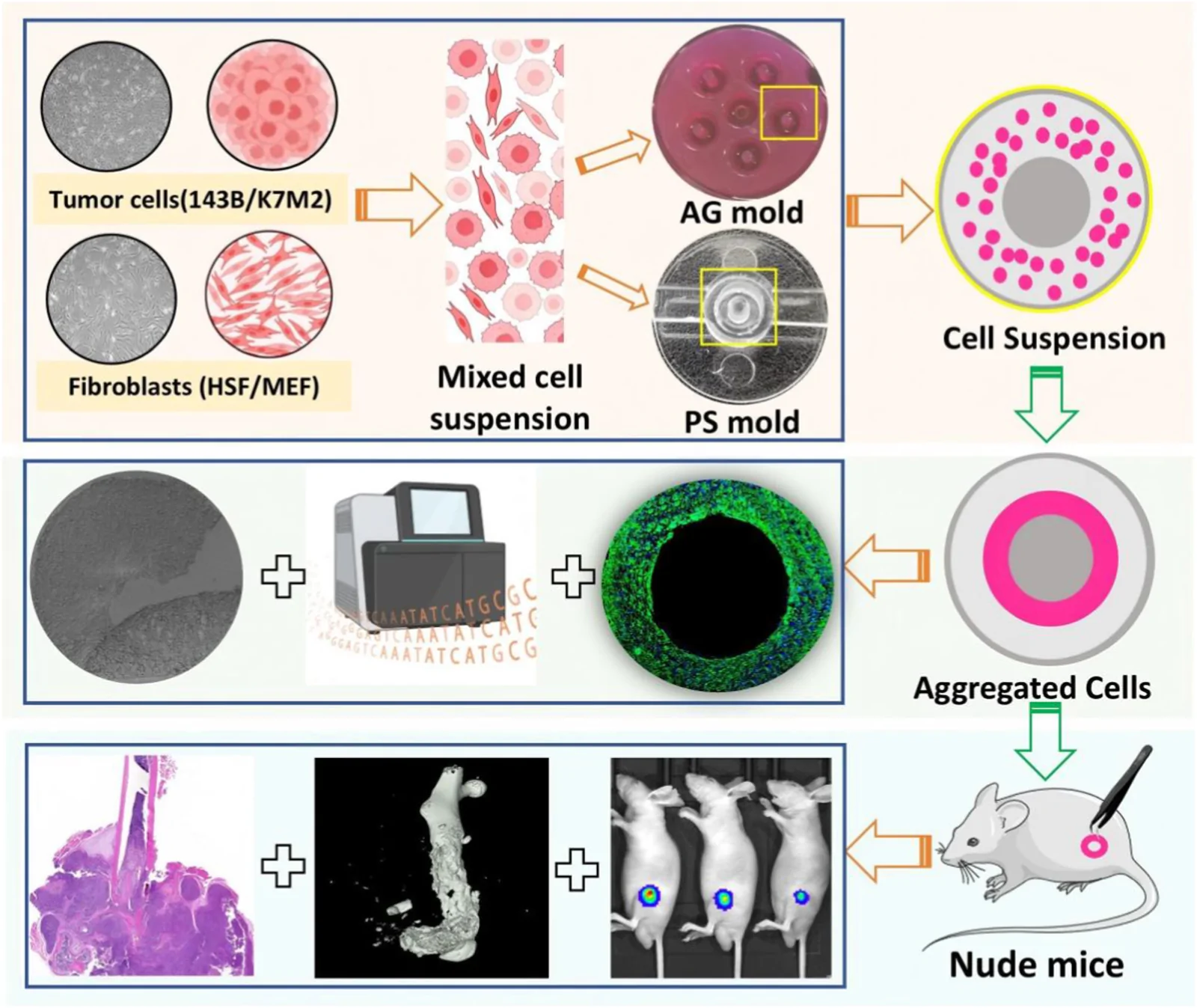
Schematic overview of the scaffold-free self-assembled cell ring organoid platform**.** Tumor cells (143B/K7M2) and fibroblasts (HSF/MEF) are mixed and seeded into agarose (AG) or polystyrene (PS) microwell molds, self-assembling into cell ring organoids within 24–48 h. The formed constructs undergo *in vitro* multi-dimensional characterization and are subsequently transplanted into nude mice for tumor modeling and *in vivo* biological evaluation.

## 2 Materials and methods

### 2.1 Cell culture

Human osteosarcoma cell line 143B and murine osteosarcoma cell line K7M2 were obtained from the American Type Culture Collection (ATCC, Manassas, VA, USA) and cultured in Dulbecco's Modified Eagle Medium (DMEM; Gibco, Thermo Fisher Scientific, Waltham, MA, USA) supplemented with 10% fetal bovine serum (FBS; Gibco) and 1% penicillin-streptomycin (Gibco). 143B cells stably expressing red fluorescent protein (143B-RFP) and human skin fibroblasts (HSF) expressing green fluorescent protein (HSF-GFP) were generated by lentiviral transduction and selected with puromycin (2 μg/mL; Sigma-Aldrich, St. Louis, MO, USA). HSF and mouse embryonic fibroblasts (MEF) were cultured in DMEM with 10% FBS. Primary soft tissue sarcoma (STS) cells were isolated from surgically resected tumor specimens from four patients (Cases S0812, S0113, S0114, and S1105) following informed consent and institutional review board approval by the Ethics Committee of Hunan Cancer Hospital (KY2022233). Primary cells were cultured in DMEM/F12 medium supplemented with 10% FBS, 1× B-27 supplement (Gibco). All cells were maintained at 37 °C in a humidified 5% CO_2_ incubator and regularly tested for mycoplasma contamination.

### 2.2 Fabrication of agarose microwell molds

Agarose (AG) microwell molds were fabricated using a polydimethylsiloxane (PDMS) template method. Sylgard 184 pre-polymer (Dow Corning, Midland, MI, USA) was mixed with curing agent at a 10:1 ratio, poured onto custom-designed micro-pillar arrays (pillar diameter 2 mm or 4 mm, height 2 mm), and cured at 80 °C for 2 h to obtain PDMS negative masters. Ultra-low gelling temperature agarose (Sigma-Aldrich) was dissolved in PBS to prepare a 2% (w/v) solution, heated to 70 °C, poured onto the PDMS masters, and allowed to gel at room temperature before demolding. Agarose microwell arrays were equilibrated in culture medium overnight at 37 °C before use. Polystyrene (PS) molds were fabricated in parallel using injection molding with identical geometric specifications; detailed dimensional parameters of both mold types are shown in Supplementary Fig. 1. For surface modification control experiments, molds were pre-treated with Anti-Adherence Rinsing Solution (StemCell Technologies, Vancouver, Canada).

### 2.3 Cell ring self-assembly

Tumor cells and fibroblasts were harvested, counted, and mixed at pre-determined ratios (fibroblast:tumor cell = 1:4 to 4:1) in culture medium. Total cell numbers were titrated from 5 × 10^5^ to 8 × 10^5^ per ring. The mixed cell suspension (70 μL/well) was seeded into agarose microwell molds. Due to the non-adherent nature of the agarose surface, cells spontaneously settled and aggregated, self-organizing into compact ring structures within 24–48 h. When using PS molds, molds were first sterilized and then pre-treated with Anti-Adherence Rinsing Solution; subsequent cell mixing, seeding volume, and self-assembly culture conditions were identical to those used for the agarose mold system.

For cell rings applied to *in vivo* transplantation, agarose molds were used for fabrication. The fibroblast-to-tumor cell ratio was fixed at 1:1, with a total of 6 × 10^5^ cells per ring. Following self-assembly, the cell rings were cultured *in vitro* for approximately 48 h before implantation.

### 2.4 Live/dead viability assay

Cell viability within rings was assessed at days 1, 3, and 5 post-fabrication using a Live/Dead Viability/Cytotoxicity Kit (Invitrogen, Thermo Fisher Scientific). Cell rings were incubated with 2 μM calcein-AM and 4 μM ethidium homodimer-1 (EthD-1) in PBS for 30 min at 37 °C. Fluorescence images were acquired using a confocal laser scanning microscope (FV3000; Olympus, Tokyo, Japan) with 4× and 10× objectives. The percentage of calcein-AM-positive (live) cells relative to total cells was calculated from three random fields per ring.

### 2.5 Histology and immunofluorescence

Cell rings and excised tumor tissues were fixed in 4% paraformaldehyde (PFA) overnight at 4 °C, dehydrated through a graded ethanol series, cleared in xylene, and embedded in paraffin. Sections (5 μm) were stained with hematoxylin and eosin (H&E), Masson's trichrome, Van Gieson, and alkaline phosphatase (ALP) using standard protocols. For immunofluorescence, deparaffinized sections underwent antigen retrieval in citrate buffer (pH 6.0) at 95 °C for 20 min, followed by blocking with 5% bovine serum albumin (BSA) for 1 h. Primary antibodies against collagen type I (COL1; 1:200; Abcam, Cambridge, UK, ab34710), α-smooth muscle actin (α-SMA; 1:200; Abcam, ab7817), and F4/80 (1:100; BioLegend, San Diego, CA, USA, 123108) were applied overnight at 4 °C. Alexa Fluor-conjugated secondary antibodies (1:500; Invitrogen) were applied for 1 h at room temperature. Nuclei were counterstained with 4′,6-diamidino-2-phenylindole (DAPI; Vector Laboratories, Burlingame, CA, USA). Images were captured using a confocal microscope (FV3000) and an automated slide scanner (VS200; Olympus).

### 2.6 Scanning electron microscopy (SEM)

Tissue specimens were fixed in 2.5% glutaraldehyde (0.1 M cacodylate buffer, pH 7.4) overnight at 4 °C, post-fixed with 1% osmium tetroxide for 1 h, dehydrated through a graded ethanol series (30%–100%), and critical-point dried using liquid CO_2_. Specimens were mounted on aluminum stubs, sputter-coated with gold-palladium, and imaged using a field-emission scanning electron microscope (SU8010; Hitachi, Tokyo, Japan) at an accelerating voltage of 5 kV.

### 2.7 Animal studies

All animal experiments were approved by the Institutional Animal Care and Use Committee of Zhejiang University (Approval No. ZJU20260796) with no restriction on sex and conducted in strict accordance with the NIH Guide for the Care and Use of Laboratory Animals. Nude mice or BALB/c mice (6–8 weeks old; SLAC Laboratory Animal Co., Shanghai, China) were housed under SPF conditions. Following adaptive housing, an orthotopic osteosarcoma model was established via lateral femoral condyle transplantation. Mice were weighed, anesthetized with isoflurane inhalation, and the surgical site was prepared and disinfected. A small incision was made on the lateral aspect of the unilateral knee joint, and the skin and subcutaneous soft tissue were sequentially separated to fully expose the lateral femoral condyle. After gently stripping the local periosteum and creating a standardized bone implantation site, the respective materials were implanted: the cell suspension group received an injection (10 μL injection volume, 5 × 10^5^ cells per mouse, in sterile PBS) and the standardized osteosarcoma organoid group received cell ring implants. Correct implant positioning was confirmed. The muscle, subcutaneous tissue, and skin incisions were then closed in layers with sutures, and the surgical site was disinfected again. Post-operatively, mice were placed in a thermostatically controlled environment for recovery and routinely monitored for mental status, activity, food intake, and incision healing.

Animals were randomly assigned to experimental groups before surgery. Three separate *in vivo* studies were conducted: (1) comparison of tumor models established via cell suspension injection versus cell-ring organoid implantation, n = 6 per group; (2) time-series observation of tumor growth and bone invasion after cell-ring organoid transplantation, with 3 mice sacrificed at each of 4 time points (total n = 12); (3) K7M2/MEF cell-ring organoid bilateral lateral femoral condyle transplantation for tumor formation evaluation, n = 14. No animals were excluded from analysis. Tumor measurement and bone imaging analysis were conducted by researchers blinded to group identities. Body weight was measured regularly, and tumor dimensions were measured twice weekly by caliper; tumor volume was calculated as V = (length × width^2^)/2. Animals were euthanized when tumor volume reached 1000 mm^3^ or at predetermined endpoints.

### 2.8 Bioluminescence imaging (BLI)

143B-Luc were used to fabricate cell rings. At day 7, 14, 21 and 28 post-transplantation, mice were intraperitoneally injected with D-luciferin (150 mg/kg; PerkinElmer, Waltham, MA, USA) and imaged using an IVIS Spectrum *in vivo* imaging system (PerkinElmer) 10 min after injection. Bioluminescent signal intensity was quantified as photons/s/cm^2^/sr using Living Image software (v4.5; PerkinElmer).

### 2.9 Micro-computed tomography (micro-CT)

At day 7, 14, 21 and 28 post-transplantation, anesthetized mice were scanned using a micro-CT system (Skyscan 1276; Bruker, Kontich, Belgium) at 50 kV and 200 μA with a 0.5 mm aluminum filter. Images were acquired at a voxel resolution of 20 μm and reconstructed using NRecon software (Bruker). Three-dimensional renderings and volumetric analyses were performed using CTAn and CTVox software (Bruker).

### 2.10 Flow cytometry

Tri-culture cell rings containing RAW264.7 macrophages were collected, washed with PBS, and digested with 0.25% trypsin-ethylenediaminetetraacetic acid (EDTA; Gibco) at 37 °C for 5–10 min. Digestion was terminated with serum-containing medium, and samples were gently pipetted into single-cell suspensions. After filtration through a 70 μm cell strainer (Corning, Corning, NY, USA) to remove undissociated aggregates, cells were collected by centrifugation at 300*g* for 5 min and washed twice with PBS. Cells were incubated with Fc receptor blocker (anti-CD16/CD32; BioLegend) at 4 °C for 15 min, followed by staining with APC-conjugated anti-mouse F4/80 antibody (1:200; BioLegend, 123116) at 4 °C for 30 min in the dark. After two PBS washes, cells were resuspended in PBS containing 1% FBS. Data were acquired using a BD FACSCanto™ flow cytometer (BD Biosciences, San Jose, CA, USA) and analyzed with FlowJo v10.8 software (Tree Star, Ashland, OR, USA).

### 2.11 Western blotting

2D-cultured HSF cells, HSF-only cell rings (3D HSF ring), and HSF+143B co-culture cell rings (3D HSF+143b ring) were collected, washed with PBS, and lysed in RIPA lysis buffer (Thermo Fisher Scientific) supplemented with protease inhibitor cocktail (Roche, Basel, Switzerland) on ice for 30 min. Lysates were clarified by centrifugation at 14,000g for 15 min at 4 °C. Protein concentrations were determined using a BCA protein assay kit (Thermo Fisher Scientific). Equal amounts of protein (30 μg/lane) were separated by 10% SDS-PAGE and wet-transferred onto PVDF membranes (Millipore, Burlington, MA, USA). Membranes were blocked with 5% non-fat milk for 1 h at room temperature and incubated overnight at 4 °C with primary antibodies against α-SMA (1:1000; Abcam, ab7817) and glyceraldehyde-3-phosphate dehydrogenase (GAPDH; 1:5000; Abcam, ab8245). After three washes with TBST, membranes were incubated with HRP-conjugated secondary antibodies (1:5000; Cell Signaling Technology, Danvers, MA, USA) for 1 h at room temperature. Chemiluminescent signals were developed using enhanced chemiluminescence (ECL) substrate (Thermo Fisher Scientific) and imaged with a ChemiDoc™ XRS + system (Bio-Rad, Hercules, CA, USA). Band intensities were quantified using ImageJ software (National Institutes of Health, Bethesda, MD, USA), and α-SMA expression levels were normalized to GAPDH.

### 2.12 Statistical analysis

All data are presented as mean ± standard deviation (SD). Comparisons between two groups were performed using two-tailed Student's t-test; comparisons among three or more groups used one-way analysis of variance (ANOVA) followed by Tukey's post hoc test. Tumor growth curves were compared by two-way repeated-measures ANOVA. All statistical analyses were conducted using GraphPad Prism 9.0 (GraphPad Software, San Diego, CA, USA). A p-value < 0.05 was considered statistically significant.

## 3 Results

### 3.1 Fabrication of scaffold-free cell ring organoids and multi-cell-source validation

To establish a standardized cell ring fabrication protocol, we designed two scaffold-free self-assembly schemes based on agarose and polystyrene microwell molds. Tumor cells (143B/K7M2) and fibroblasts (HSF/MEF) were mixed and seeded into the molds, where cells spontaneously self-organized into well-defined ring structures within 24–48 h. These ring constructs exhibited good handleability and could be directly grasped with surgical forceps and transplanted into mice without the need for Matrigel or hydrogels (Fig. 2A and B).

**Fig. 2:**
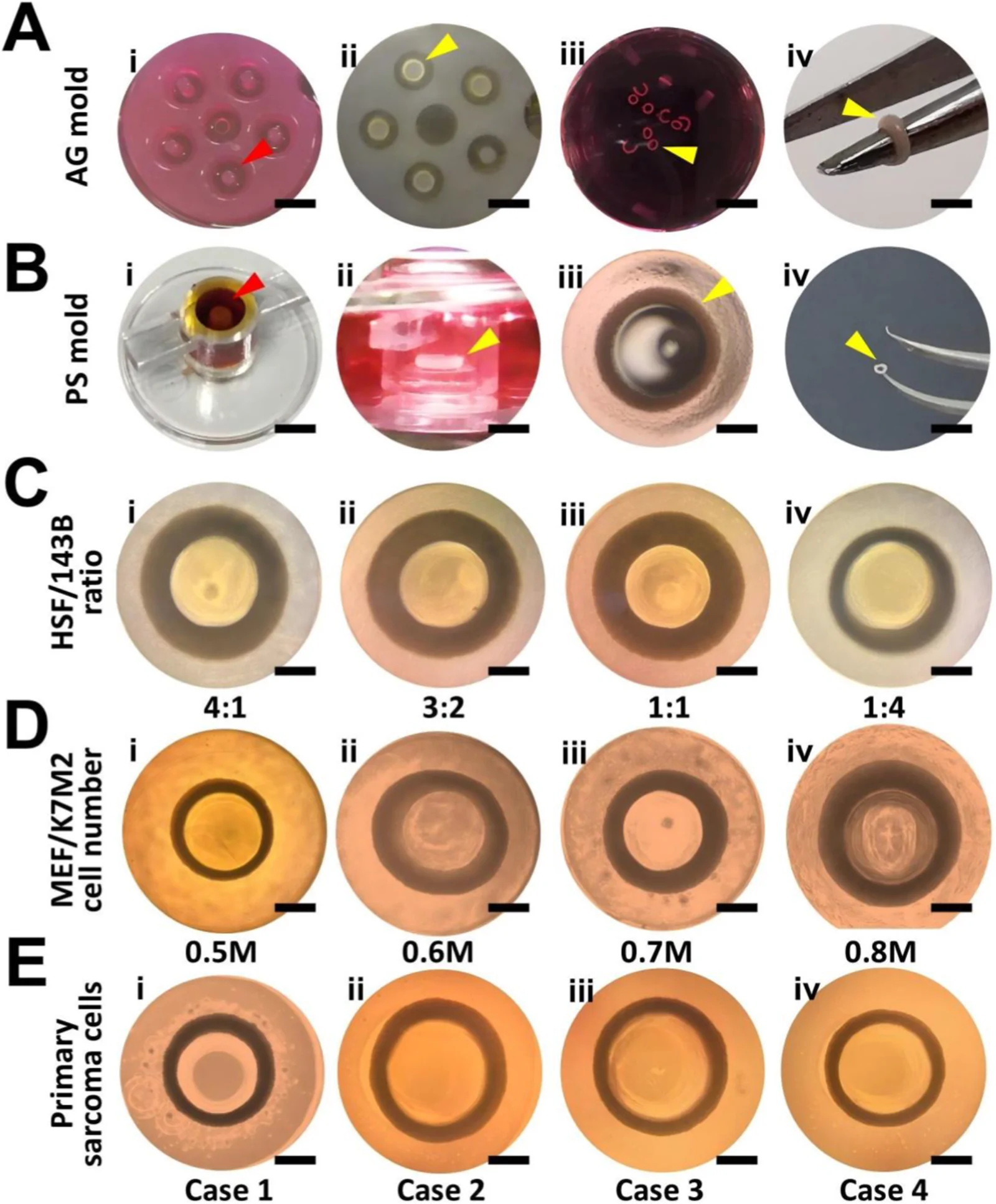
Systematic optimization of cell ring self-assembly parameters. (A) Photographs of the complete agarose (AG) mold fabrication, cell self-assembly, and forceps-mediated ring retrieval process. (B) Operational workflow for polystyrene (PS) mold molding, demolding, and ring retrieval. (C) Effect of different HSF:143B cell seeding ratios (4:1, 3:2, 1:1, 1:4) on ring morphology. (D) Ring formation outcomes of the MEF/K7M2 system under gradient total seeding densities. (E) Brightfield imaging of cell rings generated from four clinical primary soft tissue sarcoma samples. Scale bar: 2 mm.

To determine optimal fabrication parameters, we systematically examined the effects of total cell number and cell ratio on ring formation quality. The fibroblast-to-tumor cell ratio was a critical parameter: increasing the fibroblast proportion (4:1 to 4:1) significantly enhanced ring mechanical stability (Fig. 2C), attributable to the endogenous ECM proteins (e.g., collagen type I and fibronectin) secreted by fibroblasts acting as a natural biological scaffold within the rings [27]. At 0.5 million total cells, the resulting rings were translucent and structurally loose; as cell number increased to 0.6–0.8 million, ring compactness and opacity improved markedly, and the rings resisted deformation during forceps handling. To validate the cell source applicability of this platform, we extended the fabrication process to various cell systems. Mouse MEF co-cultured with K7M2 successfully formed rings (Fig. 2D). Simultaneously, four clinical primary STS samples (Cases S0812, S0113, S0114, S1105) co-cultured with HSF at a 1:1 ratio (0.6 million total cells per ring) all formed structurally intact cell rings within 48 h (Fig. 2E, Supplementary Fig. 2), confirming that this self-assembly strategy possesses cross-species adaptability.

Comparing AG and PS molds, both successfully produced cell rings; agarose, with its intrinsic anti-adhesive properties, yielded rings with smoother surfaces and more uniform contours; PS molds, which have inherent cell-adhesive properties, required pre-treatment with an anti-adherence coating to enable cell self-assembly. We further investigated the regulatory effects of mold surface modification on ring formation using PS molds (Supplementary Fig. 3): without coating treatment, the polystyrene substrate adhered dispersed cells, and neither single-cell types nor mixed cells could aggregate to form ring structures; with anti-adherence coating pre-treatment, 143B tumor cells alone still failed to self-assemble into rings, whereas pure HSF and HSF+143B co-cultures both robustly formed rings. The mechanistic basis for this phenomenon is that fibroblasts secrete collagen and other ECM components to establish physical intercellular connections, driving cell aggregation into ring structures [30]; tumor cells alone produce insufficient endogenous ECM to support 3D structure formation, necessitating the introduction of fibroblasts to provide the matrix scaffold required for scaffold-free self-assembly.

### 3.2 *In vitro* viability, structural characterization, and CAF-like transformation of co-cultured fibroblasts

To assess the impact of the self-assembly process on cell viability, we performed longitudinal Live/Dead staining on cell rings at days 1, 3, and 5 post-fabrication (Fig. 3A). Live cell proportions at all three time points exceeded 70%, indicating that cell rings overall maintained high viability within the 5-day *in vitro* culture period. The decline in viability at later time points was primarily due to progressive cell proliferation leading to cell death in the inner core region, consistent with the well-characterized phenomenon of diffusion-limited nutrient access in avascular 3D cultures [31], [32]. The outer surface regions of the rings consistently retained dense populations of viable cells, ensuring favorable contact with host tissue upon transplantation.

**Fig. 3:**
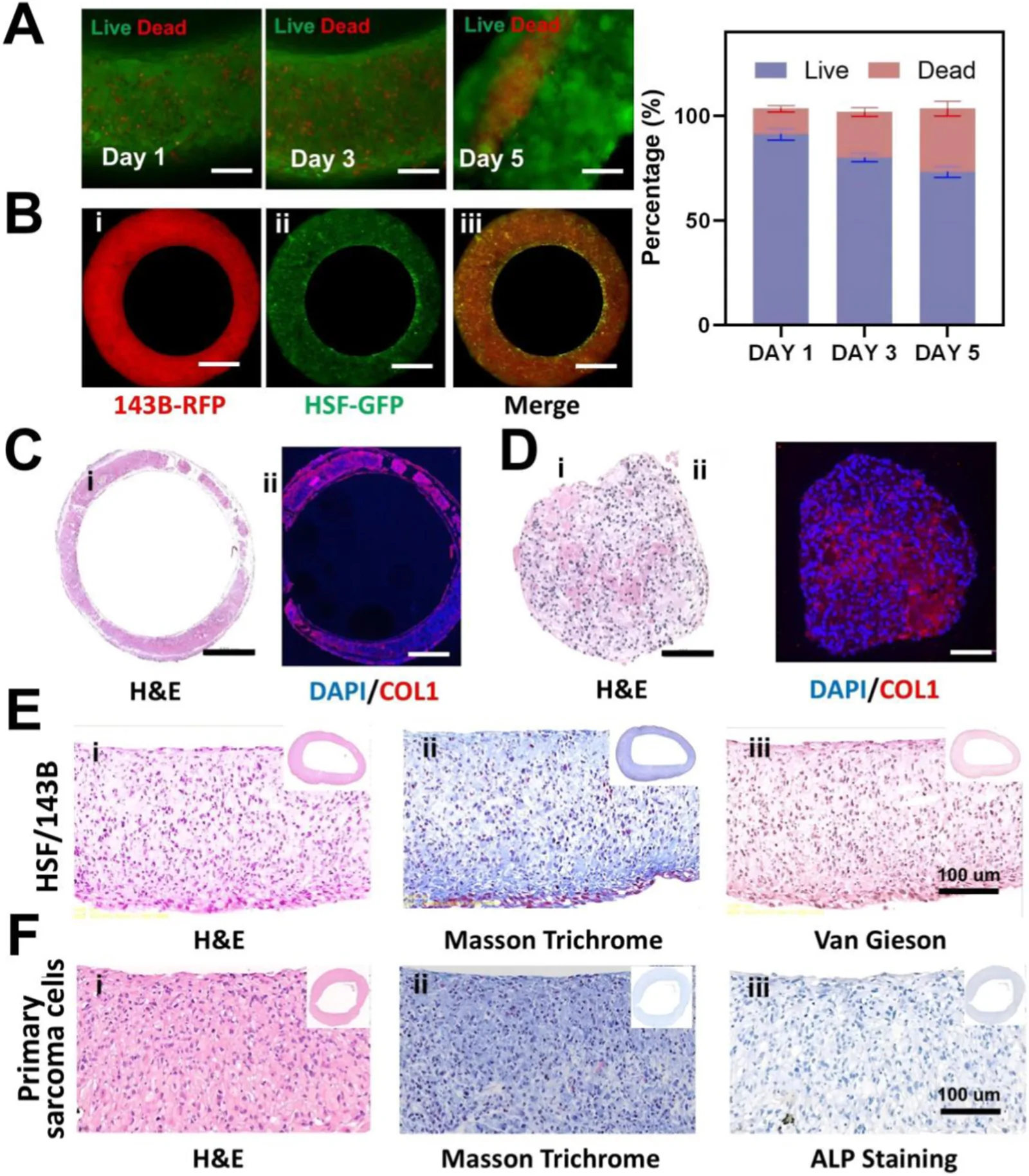
*In vitro* viability and multi-level structural characterization of cell rings. (A) Live/dead staining at different culture time points with quantitative viability analysis. (B) Confocal imaging of red fluorescent-labeled 143B tumor cells (143B-RFP) and green fluorescent-labeled fibroblasts (HSF-GFP), showing the distribution of both cell types within cell rings. (C–D) Comparative histology and COL1 immunofluorescence of ring-shaped versus solid spherical structures. (E, F) Collagen-specific staining and ALP staining of cell line-derived and primary tumor-derived cell rings, respectively. Scale bar: 100 μm.

Fluorescent tracing experiments (143B-RFP + HSF-GFP) clearly demonstrated that tumor cells and fibroblasts were interspersed throughout the ring structure, with fibroblasts forming interconnected networks enveloping tumor cell clusters (Fig. 3B), morphologically reminiscent of CAF organization in human tumor tissue [28]. H&E staining was used to compare the internal tissue architecture of ring-shaped and solid spherical 3D constructs (Fig. 3C and D): both structures contained collagen matrix deposited by fibroblasts, but the solid spherical structures exhibited extensive central necrotic regions due to impaired nutrient transport, whereas the ring structures, benefiting from the central lumen for enhanced mass exchange, showed no significant central necrosis and superior structural integrity. Masson's trichrome and Van Gieson staining of HSF/143B cell rings and clinical primary sarcoma cell rings (Fig. 3E and F) revealed extensively distributed collagen fiber networks, demonstrating that fibroblasts in the co-culture system actively secreted abundant ECM during the self-assembly phase, constructing an endogenous supportive scaffold. ALP staining of primary sarcoma-derived cell rings (Fig. 3F) revealed focal positive signals, confirming that the osteogenic differentiation potential of osteosarcoma cells was preserved under 3D culture conditions and that this *in vitro* construct retained key biological characteristics of the parental tumor.

To evaluate whether co-cultured fibroblasts underwent phenotypic transformation toward CAFs within the cell rings, we employed Western blotting and α-SMA immunofluorescence to compare protein expression profiles across three sample groups (Fig. 4): 2D-cultured human skin fibroblasts (2D HSF) showed virtually undetectable α-SMA protein expression; 3D cell rings composed solely of fibroblasts (3D HSF ring) displayed detectable but low levels of α-SMA expression, indicating that 3D mechanical stress alone can induce mild fibroblast activation; in contrast, 3D cell rings formed by co-culture of fibroblasts with 143B tumor cells (3D HSF+143b ring) exhibited markedly upregulated α-SMA protein levels, and immunofluorescence revealed extensive, high-intensity α-SMA filamentous signals. These results demonstrate that: under conventional 2D culture conditions, fibroblasts remain quiescent and do not express α-SMA; 3D spatial architecture provides mechanical stimuli that can independently drive low-level α-SMA expression and initiate fibroblast activation; the combination of tumor cell-fibroblast paracrine crosstalk and the 3D mechanical microenvironment further strongly promotes high-level α-SMA expression. In other words, the cell ring system accomplishes stromal cell pre-activation during the *in vitro* culture phase, establishing a pro-tumorigenic stromal microenvironment prior to transplantation. Upon implantation *in vivo*, these pre-activated fibroblasts can directly participate in ECM remodeling and regulate tumor proliferation and invasion, representing a key mechanism underlying the superior engraftment efficiency of cell rings compared with conventional single-cell suspension transplantation [28], [29].

**Fig. 4:**
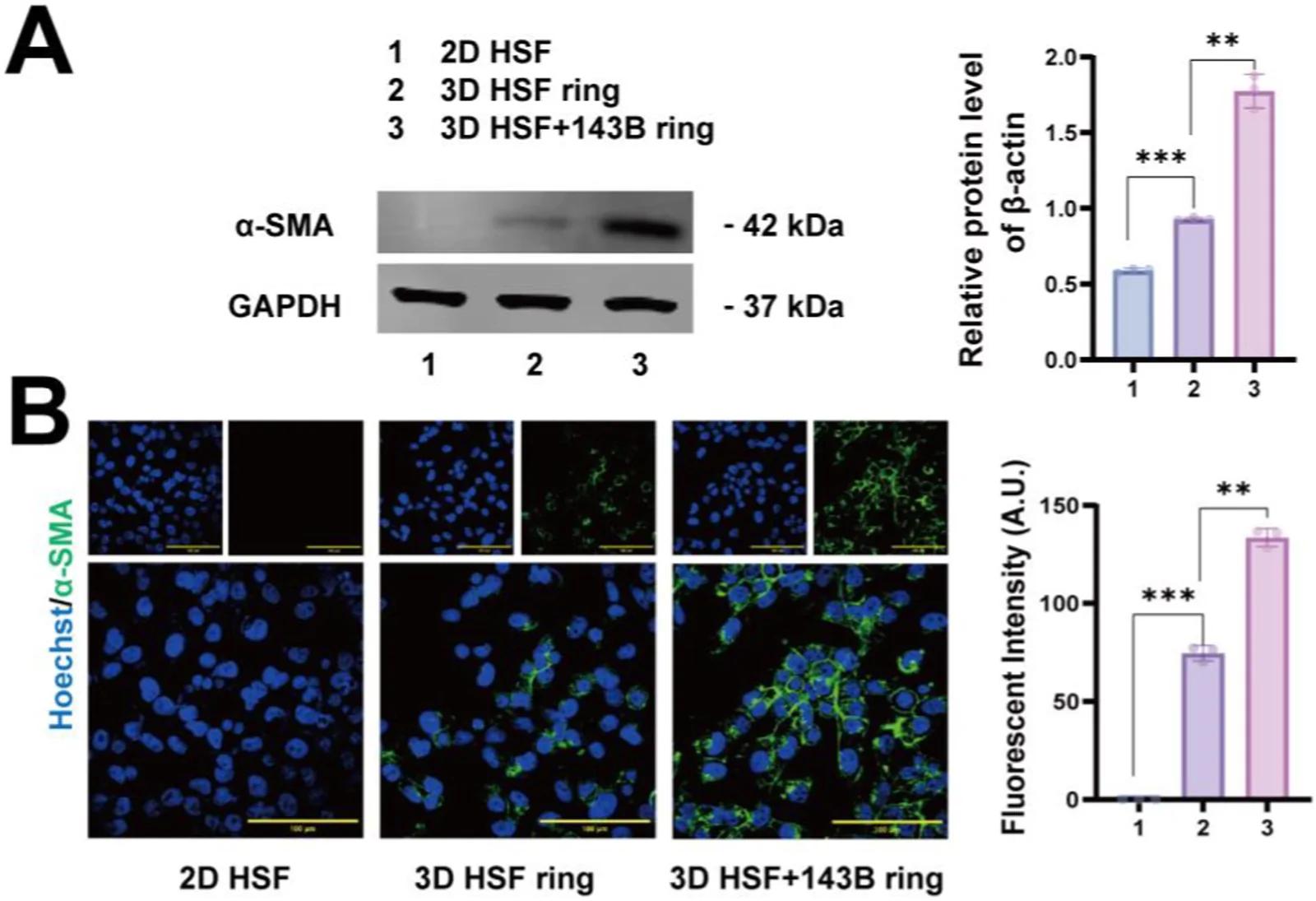
CAF-like phenotypic transformation of fibroblasts within cell rings. (A) Western blot detection of α-SMA protein expression in 2D-cultured fibroblasts (2D HSF), fibroblast-only 3D rings (3D HSF ring), and fibroblast-143B co-culture 3D rings (3D HSF+143B ring). Relative α-SMA expression was quantified by semi-quantitative densitometry. (B) Immunofluorescence staining for α-SMA. Semi-quantitative quantification of α-SMA fluorescence intensity was carried out for statistical comparison of α-SMA expression levels. Data are presented as mean ± SD. Student's t-test was used for statistical analysis. **p < 0.005; ***p < 0.001.

### 3.3 Transcriptomic validation of the molecular fidelity of cell ring organoids to original tumors

To systematically evaluate the transcriptomic similarity between *in vitro* constructs and clinical native tumor tissues, we performed bulk RNA sequencing on 2D-cultured cells, cell line-derived cell ring organoids, primary cell ring organoids, and patient tumor specimens. Principal component analysis (PCA, Fig. 5A) revealed clear sample group separation: traditional 2D-cultured cells and clinical tumor samples clustered at considerable distance from each other; in contrast, both cell line-derived and primary cell ring organoids fell within the distribution range of native tumor tissues, demonstrating that the 3D self-assembled ring structure reshaped the global gene expression profile and could recapitulate the transcriptional characteristics of *in vivo* tumors.

**Fig. 5:**
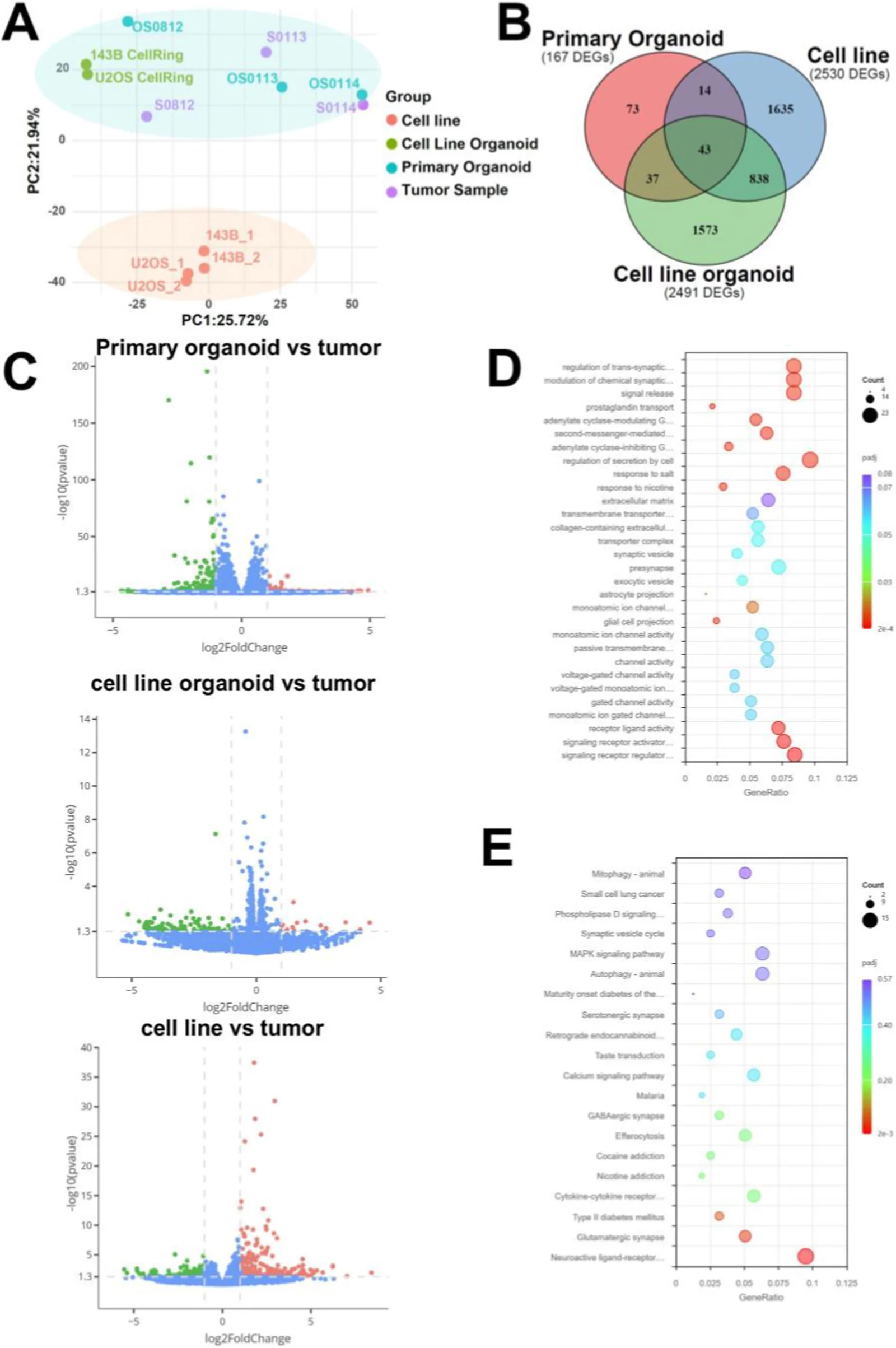
Transcriptomic analysis validating the fidelity of cell ring organoids to original tumor features. (A) PCA clustering analysis. (B) Venn diagram of differentially expressed genes. (C) Volcano plots of differential gene expression for three pairwise comparisons. (D, E) GO and KEGG enrichment analyses identifying key differentially regulated biological functions and signaling pathways.

Venn diagram quantitative analysis of DEGs (Fig. 5B) further quantified the degree of transcriptional deviation among the three model groups: all three *in vitro* systems exhibited DEGs relative to tumor tissue due to detachment from the intact *in vivo* tissue microenvironment, but the number of unique DEGs directly reflects model fidelity. Because cell lines, as immortalized passaged cells, have already diverged considerably from the original tumor phenotype, we observed that both cell ring organoids and cell lines shared many DEGs relative to tumor tissue; in contrast, primary cell ring organoids showed the lowest total DEG count and the fewest specific transcriptional perturbations, with the highest concordance with native tumor gene expression patterns.

Volcano plot results from pairwise comparisons with clinical tumor tissues showed that comparisons involving cell lines alone versus tumor tissue yielded large numbers of significantly up- or down-regulated genes (Fig. 5C). This indicates that cell ring organoids can preserve the vast majority of native tumor transcriptional features, whereas prolonged 2D culture causes large-scale transcriptional drift, with cellular gene expression patterns progressively deviating from the *in vivo* tumor phenotype.

Subsequent Gene Ontology (GO) functional enrichment analysis of DEGs between cell lines alone and cell ring organoids (Fig. 5D) identified top-ranked terms including synaptic remodeling, ECM assembly, ion channel transport, glial cell process formation, and receptor-ligand signaling regulation. These functional clusters directly reflect the core phenotypic differences between 3D multicellular structures and dispersed monolayer cells: cell ring organoids can form intercellular junctions and deposit endogenous collagen matrix, establishing electrochemical microenvironments through various ion channels; dispersed monolayer cells lose spatial cell-cell contacts and the capacity for structured ECM secretion, with these biological functional modules being generally downregulated.

Kyoto Encyclopedia of Genes and Genomes (KEGG) pathway enrichment analysis (Fig. 5E) identified multiple key differentially regulated signaling pathways: mitophagy and general autophagy pathways were significantly enriched, attributable to nutrient and oxygen gradients within the 3D ring structure that activate adaptive autophagic responses, whereas the homogeneous monolayer culture environment does not elicit such stress responses; additionally, calcium signaling, mitogen-activated protein kinase (MAPK) signaling, various neuronal synapse pathways, and cytokine-receptor interaction pathways were also significantly enriched, representing pathways responsible for regulating multicellular communication, matrix remodeling, and cellular stress adaptation within the TME. Collectively, these transcriptomic results confirm that the scaffold-free cell ring organoid platform developed in this study can restore the core transcriptional programs of native tumors, exhibiting stronger TME recapitulation capacity compared with traditional monolayer cell suspensions; furthermore, they provide molecular-level explanations for the superior *in vivo* tumor formation outcomes observed after cell ring organoid transplantation [20], [33].

### 3.4 *In vivo* transplantation: cell ring organoids exhibit significantly higher engraftment efficiency and tumor uniformity compared with cell suspensions

We next functionally validated *in vivo* tumor formation efficacy. Two *in vivo* modeling approaches—traditional cell suspension injection and surgical transplantation of cell ring organoids—were compared. The overall experimental timeline is shown in Fig. 6A: the experimental cycle encompassed cell expansion, cell ring organoids fabrication, orthotopic mouse transplantation, *in vivo* imaging monitoring, and endpoint micro-CT scanning with histological analysis. Fig. 6B visually illustrates the technical pathway differences between the two transplantation methods. Fig. 6C documents the practical procedures of cell ring *in vitro* harvesting and intraoperative implantation; the cell ring organoids possessed sufficient mechanical strength to be manipulated with surgical forceps for grasping and site-directed implantation, making the transplantation procedure simple and controllable. BLI was employed for longitudinal assessment of post-transplantation *in vivo* cell retention and early proliferation (Fig. 6D). At day 14 post-transplantation, BLI signals in the cell ring group were concentrated and restricted to the implantation site, whereas signals in the suspension group were diffusely dispersed, demonstrating that the cell ring organoids can prevent the loss and dispersal of free cells associated with fluid-mediated washout. Long-term tumor growth curves are presented in Fig. 6E: tumors in the cell ring organoids group entered the detectable phase earlier, with tumor growth observable as early as days 5–7 post-transplantation, whereas the cell suspension group generally exhibited prolonged engraftment latency of 10–14 days; at all subsequent detection time points, tumor volumes in the cell ring organoids group were significantly greater than those in the suspension group. Multiple independent replicate experiments confirmed that the cell ring organoids group showed superior engraftment rates and intra-group tumor growth synchrony compared with traditional suspension-based modeling; endpoint tumor weight and volume statistics (Fig. 6F and G) yielded fully consistent conclusions. Intra-group coefficient of variation (CV) analysis demonstrated that tumor volume fluctuation among individual animals in the cell ring organoids group was substantially smaller than in the suspension group, indicating that animal models constructed using this method exhibit stronger tumor uniformity, which can reduce the number of animals required per experiment, in alignment with the 3R principles [34], [35].

**Fig. 6:**
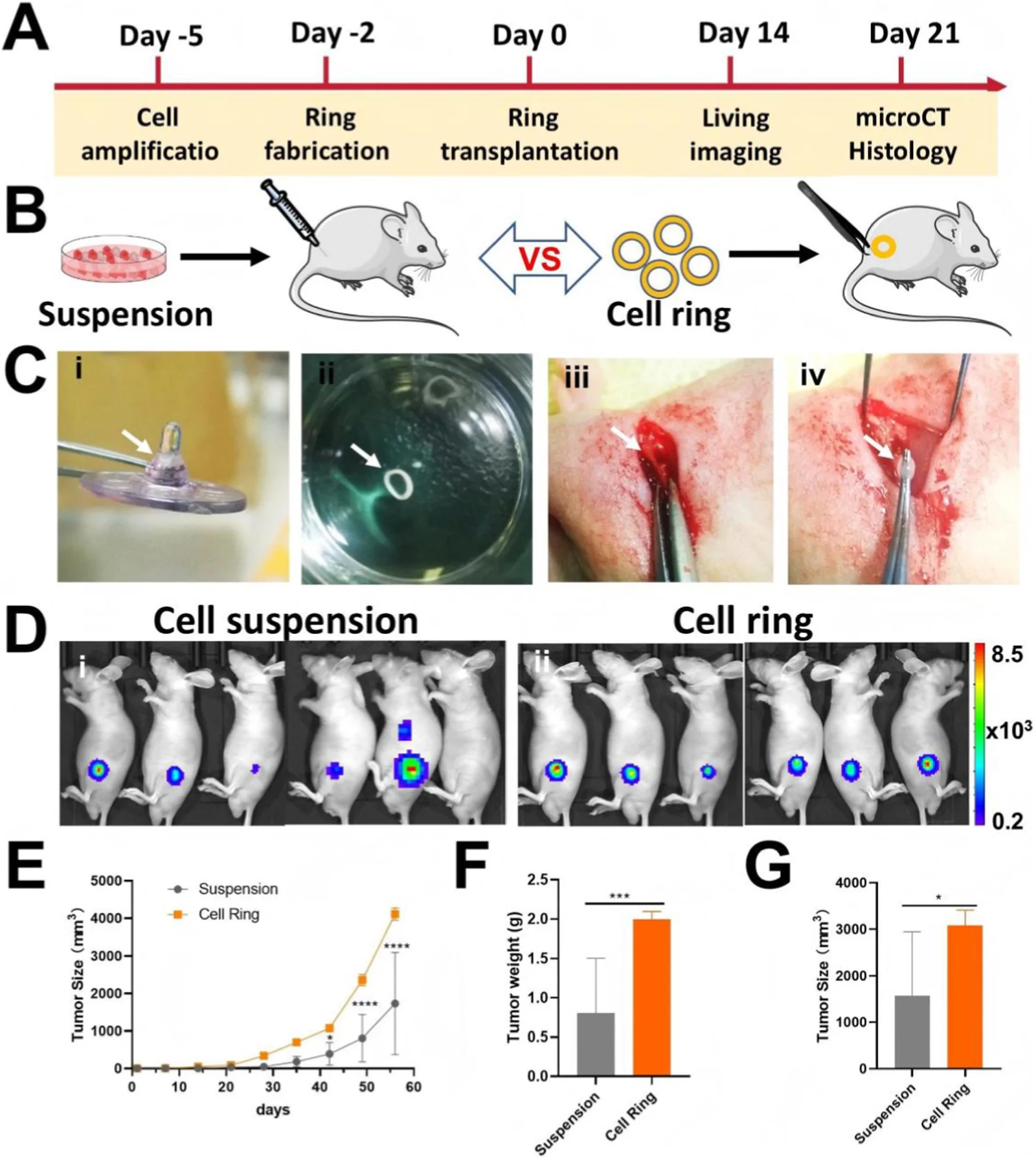
Dynamic monitoring and quantitative analysis of *in vivo* tumor formation by cell rings**.** (A) Experimental timeline. (B) Schematic diagram of the two transplantation strategies. (C) Intraoperative photographs of cell ring manipulation. (D) *In vivo* bioluminescence imaging comparing tumor growth between the two groups. (E–G) Quantitative statistics of tumor growth curves, endpoint tumor weights, and micro-CT-measured tumor volumes, respectively. Data are presented as mean ± SD. Student's t-test was used for statistical analysis. *p < 0.05; ***p < 0.001.

To further validate cross-species applicability, we employed the mouse osteosarcoma cell line K7M2 co-cultured with MEF at a 1:1 ratio to prepare cell ring organoids and performed lateral femoral condyle transplantation verification in immunocompetent mice (Supplementary Fig. 4). The results showed that MEF/K7M2 cell ring organoids similarly exhibited high engraftment rates and rapid growth characteristics *in vivo*, further supporting the cross-species applicability of this platform, and demonstrating that immunocompetent normal mice can be used for tumor modeling with our cell ring organoids.

### 3.5 Cell ring organoids exhibit enhanced bone invasion capacity and pathological features closely recapitulating clinical osteosarcoma

To further evaluate the application value of cell ring organoids in orthotopic osteosarcoma models, we assessed the bone tissue invasion patterns during tumor progression (Fig. 7A–C). BLI signals in the cell ring organoids group exhibited sustained and significant increases over time, and micro-CT 3D reconstructions revealed that ring-derived tumors displayed a characteristic osteolytic growth pattern, with tumors invading the femoral cortex and extending into the medullary cavity, accompanied by focal trabecular bone loss and periosteal reaction. Moreover, this bone-invasive capacity was further intensified with increasing tumor volume (Fig. 7D and E). Histological H&E staining further confirmed (Fig. 7F) that ring-derived tumor tissues contained abundant osteoid matrix deposition, tumor bone formation, and active osteoclasts—pathological features highly consistent with clinical osteosarcoma patient biopsy findings [36], [37]. In comparison, suspension-derived tumors showed substantially weaker bone destruction and tumor bone matrix formation.

**Fig. 7:**
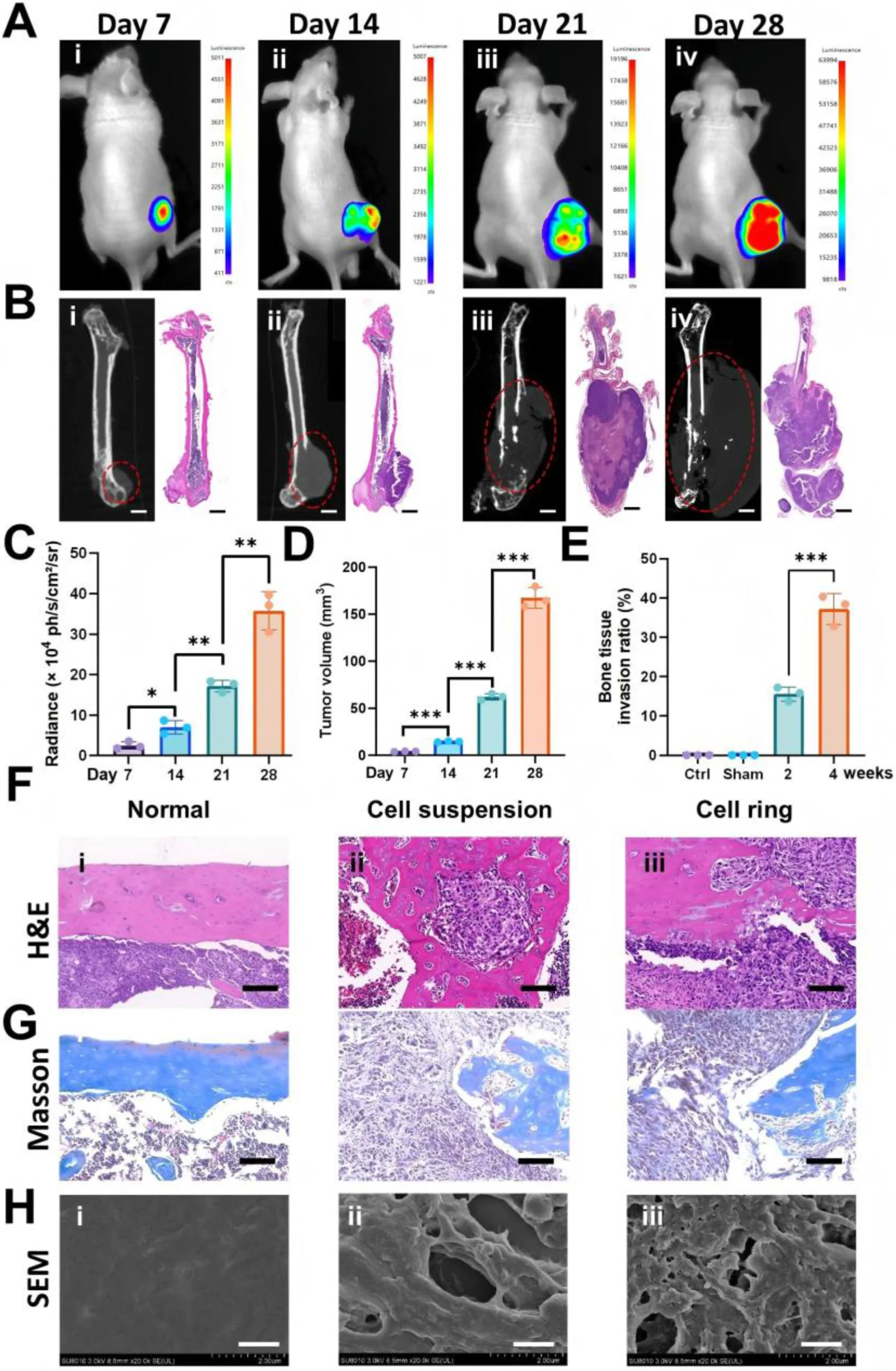
Assessment of tumor invasion capacity in the cell ring orthotopic osteosarcoma model. (A) *In vivo* bioluminescence imaging at days 7, 14, 21, and 28 post-transplantation. (B) Micro-CT bone scans and corresponding femoral tissue H&E sections demonstrating tumor infiltration and bone destruction. (C–E) Quantitative statistics of tumor fluorescence radiant efficiency, tumor volume, and bone invasion proportion at different time points. (F–H) H&E staining, Masson's trichrome staining, and scanning electron microscopy (SEM) comparing tumor matrix structure, bone destruction, and extracellular microscopic morphology among normal bone tissue, cell suspension group, and cell ring group. Scale bar: 100 μm. Data are presented as mean ± SD. Unpaired Student's t-test was used. *p < 0.05; **p < 0.01; ***p < 0.001.

Masson's trichrome staining demonstrated extensive blue-stained collagen fiber deposition within ring-derived tumors, and SEM further confirmed their dense fibrillar ECM network architecture (Fig. 7G and H). These pathological features—including tumor osteoid matrix production, osteoclast activation, and abundant collagenous stroma—closely mirror clinical osteosarcoma biopsy pathology, further validating the superiority of the cell ring platform in recapitulating osteosarcoma clinical pathological characteristics [37], [38].

Tumor-associated macrophages (TAMs) are important components of the osteosarcoma microenvironment, playing key roles in tumor progression, bone destruction, and drug resistance [39]. To explore the potential of the cell ring organoids platform for immuno-oncology research, we co-seeded RAW264.7 mouse macrophages together with K7M2 osteosarcoma cells and MEF fibroblasts into agarose microwell molds, conducting an exploratory tri-culture self-assembly study (Supplementary Fig. 5). Flow cytometric analysis showed that RAW264.7 macrophages could be successfully integrated into the cell ring organoids; at culture day 2, the proportion of F4/80-positive macrophages within organoids reached 11.7%, and immunofluorescence visualized extensive macrophage infiltration into the cell ring matrix. By culture day 4, the macrophage proportion decreased to 3.5%, with markedly reduced numbers of infiltrating cells, reflecting the gradual loss of macrophages during prolonged *in vitro* culture due to factors such as matrix barriers and changes in chemokine gradients. These preliminary results indicate that the cell ring organoids platform has the potential to incorporate immune cells and construct a more complete tumor immune microenvironment, providing a new experimental tool for subsequent immuno-oncology applications.

## 4 Discussion

This study constructed and systematically characterized a scaffold-free self-assembled cell ring organoids tumor modeling platform. Leveraging the intrinsic self-organizing properties of tumor cell-fibroblast co-culture systems, this platform spontaneously forms 3D organoid structures that are rich in endogenous ECM, structurally stable, and directly amenable to surgical implantation, without reliance on exogenous matrix gels or hydrogels. Compared with traditional single-cell suspension transplantation models, the cell ring organoids system achieves higher engraftment rates, faster tumor growth kinetics, and superior intra-group tumor uniformity, effectively reducing experimental animal usage and decreasing experimental bias, in significant alignment with the core principles of the 3Rs [16], [17].

Current mainstream organoid culture systems generally rely on exogenous matrices such as Matrigel or collagen to support structural formation. Although these satisfy *in vitro* expansion requirements, exogenous matrices have complex compositions, significant batch-to-batch variability, and residual growth factors that readily confound drug sensitivity testing results, severely compromising experimental reproducibility and result reliability [22], [23], [40], [41]. In contrast, this study employs endogenously deposited ECM secreted by co-cultured fibroblasts to replace traditional exogenous scaffolds.

We further systematically benchmark our cell-ring organoid platform against other prevailing 3D tumor models. First, compared with conventional spheroids: typical spheroids are limited to a diameter of 100–200 μm and cannot meet the demand for large-scale sarcoma modeling. Once solid spheroids exceed approximately 500 μm in diameter, limited diffusion of nutrients and oxygen inevitably leads to extensive central necrosis. The hollow ring architecture of our constructs keeps all regions within a critical diffusion limit. Second, histological and ultrastructural verification confirmed that this endogenous ECM is orderly in arrangement and rich in composition, highly resembling the native tumor stromal microenvironment, endowing the platform with unique application advantages for desmoplastic tumor modeling and ECM-targeted drug evaluation [42].

The superior *in vivo* tumor formation performance of the cell ring organoids system arises from the synergistic regulation of multiple biological mechanisms. First, cell rings already possess mature cell–cell and cell–ECM connections formed *in vitro*; after transplantation, they do not need to undergo the single-cell re-aggregation process, substantially shortening the time window during which tumor cells are exposed to the risk of anoikis and effectively improving transplanted cell survival efficiency [43]. Second, fibroblasts within the co-culture system can be pre-activated to the CAF-like phenotype during the 3D self-assembly process, expressing high levels of α-SMA and continuously providing paracrine signals, thereby constructing a pre-activated pro-tumorigenic stromal microenvironment prior to transplantation. Upon implantation *in vivo*, these cells can immediately participate in the tumor remodeling process, circumventing the protracted process of host fibroblast recruitment and activation required by traditional suspension transplantation [28], [29]. Finally, the intact and compact ring-shaped physical structure anchors cells at the implantation site, significantly reducing post-transplantation cell dispersion and fluid-mediated washout loss, effectively addressing the key problems of low cell retention and unstable engraftment observed in traditional suspension and PDX models [44].

The *in vivo* BLI results further visually corroborated this advantage, demonstrating that cell ring organoids can significantly enhance *in vivo* cell retention efficiency. Stable cell retention and engraftment capacity not only shorten tumor latency but also markedly reduce the intra-group CV of tumor volume, substantially improving model uniformity and statistical testing power, enabling precise detection of subtle drug efficacy differences with smaller animal sample sizes, better suited for standardized preclinical drug screening studies [45], [46].

Transcriptomic analysis provided molecular-level systematic confirmation of the high fidelity of the cell ring organoids platform in recapitulating native tumor phenotypes. Monolayer 2D-cultured cells exhibited large numbers of DEGs relative to clinical tumor tissues, with the most pronounced transcriptional deviation. In contrast, cell ring organoids formed through 3D self-assembly effectively corrected gene expression abnormalities induced by monolayer culture, significantly narrowing the transcriptional gap with native tumors, with primary sarcoma-derived cell rings showing the highest similarity of gene expression profiles to clinical tumor tissues. This progressive molecular repair pattern convincingly demonstrates that the 3D spatial architecture and fibroblast co-culture microenvironment can maintain the intrinsic biological characteristics of tumor cells, avoiding the phenotypic drift induced by 2D culture, and enabling *in vitro* models to closely recapitulate the *in vivo* tumor state at the molecular level [20], [33]. On this basis, this study further validated the unique value of cell ring organoids in osteosarcoma-specific pathological simulation through the femoral orthotopic transplantation model: ring-derived tumors accurately recapitulated the characteristic clinical osteosarcoma pathological features of cortical bone invasion, medullary cavity infiltration, trabecular bone destruction, and tumor osteoid matrix deposition, whereas traditional suspension transplantation only formed localized superficial lesions with significantly insufficient bone destruction and tumor matrix remodeling capacity, indicating that this platform is better suited for investigating osteosarcoma bone invasion mechanisms and for *in vivo* evaluation of anti-bone-destruction agents [36], [37].

The exploratory immune co-culture experiments in this study further expand the application boundaries of the platform. Traditional tumor organoids generally lack immune cell components, making it difficult to simulate the complete tumor immune microenvironment and severely limiting immuno-oncology research. This study, through flow cytometry and immunofluorescence, confirmed that RAW264.7 macrophages can be stably integrated into the 3D cell ring structure, demonstrating that this scaffold-free self-assembly system possesses the capacity to accommodate immune cells and construct multi-cellular composite tumor models, overcoming the key shortcoming of immune component deficiency in traditional organoids. In the future, additional immune cell subsets—such as T cells, natural killer (NK) cells, and myeloid-derived suppressor cells (MDSCs)—could be introduced to construct a more complete tumor immune microenvironment model, providing an experimental tool with greater physiological relevance for osteosarcoma immune mechanism research and immunotherapeutic drug screening [47], [48].

Simultaneously, the successful construction and *in vivo* tumor formation of K7M2 and MEF mouse cell ring organoids confirm that this technology possesses good cross-species applicability. From an animal ethics perspective, the platform, with its consistently high engraftment rates and high model uniformity, achieves reduction in experimental animal numbers (Reduction) and shortens the duration of tumor burden borne by animals through reduced engraftment latency (Refinement), in alignment with the core requirements of the 3R principles. Furthermore, this standardized modeling system can establish a tiered research paradigm of “*in vitro* organoid primary screening followed by *in vivo* precision validation,” effectively reducing the number of compounds entering the *in vivo* testing phase and further enhancing the scientific validity and ethical compliance of animal experiments [49].

Several limitations of this study should be acknowledged. First, the transcriptomic analysis involved limited numbers of clinical samples and cell samples; future studies should expand sample sizes to further enhance the generalizability of conclusions. Second, technical validation in this study primarily focused on osteosarcoma and STS; the modeling applicability of this platform in other solid tumor types remains to be systematically investigated. Third, this study only characterized model molecular features at the transcriptomic level, lacking cross-validation from proteomic, metabolomic, and other multi-omics data; future multi-omics integrated analyses will more comprehensively elucidate the molecular similarity between cell ring organoids and native tumors. Fourth, the RAW264.7 macrophage tri-culture system remains at the *in vitro* exploratory stage, and its *in vivo* immune microenvironment construction and functional validation await further investigation. Fifth, vascularization of the *in vivo* tumor grafts was not explored in the current work. Future work can further investigate whether cell ring organoids confer advantages in vascularization compared with conventional cell suspension tumor models. Sixth, HSF were selected as the stromal component for *in vitro* tri-culture. Although HSF support ring self-assembly and ECM deposition in our system, they are not tissue-matched stromal cells such as bone marrow-derived mesenchymal stromal cells or primary osteosarcoma-associated fibroblasts. The fibroblast origin may affect ring morphogenesis, ECM production, and CAF-like activation. Definitive confirmation of CAF identity requires combinatorial analysis of multiple markers including FAP, PDGFRα and podoplanin, which we plan to perform in future work to fully characterize the activation status of stromal cells. Future studies are warranted to evaluate potential differences when using tissue-matched stromal cells.

In summary, the scaffold-free self-assembled cell ring organoids platform established in this study overcomes multiple bottlenecks of traditional tumor modeling technologies, possessing the multiplex advantages of high engraftment rates, high uniformity, high pathological fidelity, and the capacity for immune component integration. It provides a standardized, optimizable, and ethically compliant novel modeling strategy for mechanistic research and preclinical drug screening across a broad spectrum of solid tumors, such as osteosarcoma and soft tissue sarcoma. Through subsequent system optimization and multi-dimensional validation, this platform is expected to further expand its application value in basic tumor research and clinical translation.

## 5 Conclusions

This study reports a novel animal tumor modeling platform based on scaffold-free self-assembled cell ring organoids. The technology requires no exogenous matrices or hydrogels, producing ring-shaped organoids with endogenous ECM support through co-culture self-assembly of tumor cells and fibroblasts, achieving consistently high engraftment rates *in vivo*. Tumor growth kinetics, uniformity, and TME fidelity were all significantly superior to those of traditional cell suspension methods. Western blotting and immunofluorescence confirmed that co-cultured fibroblasts underwent CAF-like phenotypic transformation (α-SMA upregulation) within the organoids, and transcriptomic analysis provided molecular-level validation of the platform's ability to preserve original tumor gene expression features. The lateral femoral condyle orthotopic transplantation model demonstrated that ring-derived tumors possess stronger bone invasion capacity and clinical osteosarcoma pathological features; flow cytometry and immunofluorescence verified the successful integration of RAW264.7 macrophages into the cell ring organoids. This method is applicable to tissue cells from different species, effectively reduces animal consumption in alignment with the 3R principles, and may provide a potential platform for preclinical drug screening, tumor-stroma interaction studies, and personalized tumor model establishment.

## CRediT authorship contribution statement

**Zhicheng Tong:** Writing – original draft, Visualization, Investigation, Formal analysis. **Shuo Yang:** Investigation, Data curation. **Jiayi Chen:** Writing – original draft, Visualization. **Haidong Guo:** Methodology, Investigation. **Piqian Zhao:** Resources, Project administration. **Hongwei Wu:** Writing – review & editing, Supervision, Methodology, Conceptualization.

## Ethics and animal experiments

The clinical specimens were obtained with the patients' informed consent and were approved by the Ethics Committee of Hunan Cancer Hospital (KY2022233). All animal experiments were approved by the Zhejiang University Laboratory Animal Welfare and Ethical Review Committee (No. ZJU20260796).

## Disclosure of AI tool utilization

During the preparation of this manuscript, AI tools were employed solely for language polishing, including sentence refinement and grammar correction. They were not involved in any academic creation processes of the study, such as the formulation of core viewpoints, research design, data collection and analysis, or conclusion derivation.

## Funding

This study was funded by the Key Project for Scientific and Technological of the National Administration of Traditional Chinese Medicine ,GZY-KJS-ZJ-2026-075, and the 10.13039/501100004731Zhejiang Provincial Natural Science Foundation (No. TGD24H160005).

## Declaration of competing interest

The authors declare that they have no known competing financial interests or personal relationships that could have appeared to influence the work reported in this paper.

## Data Availability

Data will be made available on request.
