# Supplementary material for: Scaffold-free self-assembled cell ring organoids enable efficient tumor modeling with high engraftment rates, superior tumor uniformity, and reduced animal consumption
Source: Mater Today Bio. 2026 Nov 2;41:103790. doi: 10.1016/j.mtbio.2026.103790 (PMC13756348; doi:10.1016/j.mtbio.2026.103790)
Supplement: Supplementary file 1 [file mmc1.docx]

**Scaffold-Free Self-Assembled Cell Ring Organoids Enable Efficient Tumor Modeling with High Engraftment Rates, Superior Tumor Uniformity, and Reduced Animal Consumption**

# Supplementary Figure Legends

**Supplementary Figure 1. Specifications and photographs of AG and PS molds.**

Top and side views of AG and PS molds.

**Supplementary Figure 2. Photographs of primary soft tissue sarcoma cell rings.**

Images of cell rings derived from four primary STS cases (S0812, S0113, S0114, S1105). Scale bar: 100 μm.

**Supplementary Figure 3. AG mold coating comparison experiment.**

Side-view comparison images and summary table of ring formation under uncoated (w/o coating) and anti-adherence-coated (with coating) AG mold conditions for HSF alone, 143B alone, and HSF+143B co-cultures. Scale bar: 2 mm.

**Supplementary Figure 4. In vivo bioluminescence images of tumor formation by K7M2:MEF mouse cell rings.**

*In vivo* bioluminescence images following transplantation of cell rings formed by K7M2 mouse osteosarcoma cells co-cultured with MEF mouse embryonic fibroblasts at a 1:1 ratio.

**Supplementary Figure 5. Exploratory study of RAW264.7 macrophage integration into cell ring organoids.**

(A) Flow cytometric quantification of F4/80-positive macrophage proportions at different culture durations. (B) Immunofluorescence verification of macrophage distribution. Scale bar: 100 μm.
